# Family meal frequency and its association with food consumption and nutritional status in adolescents: A systematic review

**DOI:** 10.1371/journal.pone.0239274

**Published:** 2020-09-18

**Authors:** Giselle Rhaisa do Amaral e Melo, Priscila Olin Silva, Jennifer Nakabayashi, Mariane Viana Bandeira, Natacha Toral, Renata Monteiro

**Affiliations:** Department of Nutrition, University of Brasilia, Brasilia, Distrito Federal, Brazil; Instituto Federal Goiano, BRAZIL

## Abstract

This systematic review evaluated the association between frequency of family meals (FFM) and nutritional status (NS) and/or food consumption (FC) in adolescents. The protocol was registered with PROSPERO (CRD42017062180) and followed the Preferred Reporting Items for Systematic Reviews and Meta-Analyses guidelines. No publication date, language, or meal type restrictions were imposed. Only full-text original articles were included; qualitative studies were excluded. Studies were identified by searching 5 electronic databases (PubMed, Web of Science, Scopus, BVS Brazil, and Adolec) and gray literature (Google Scholar) and by scanning reference lists of included articles. Risk of bias was assessed using the Newcastle-Ottawa scale for cohort and cross-sectional studies. Initial search yielded 2001 results and 47 articles were included. An updated literature search added 3 articles. Of the 50 studies included, 25 studied the association between FFM and NS, 32 investigated the association between FFM and FC, being that seven studies analyzed both outcomes. Thirty-four were cross-sectional studies, 12 were longitudinal studies, and 4 studies analyzed both cross-sectional and longitudinal data. Thirty-five studies were rated as having good quality, whereas 19 were of fair quality. Sample size ranged from 140 to 102 072 participants. Most investigations evaluated the frequency of breakfast, lunch, and/or dinner/supper/evening meals over a 1-week period. Seventeen studies identified a positive relationship between high FFM and better NS, and 26 found a positive association between high FFM and better FC. In conclusion, this review showed an association between FFM and healthy dietary patterns, such as increased consumption of fruits and vegetables. Further research is needed to understand the association between FFM and NS, since some studies showed a protective role of family meals against obesity in this age group, whereas other studies identified no significant association between these variables.

## Introduction

Eating habits that tend to involve large amounts of ultra-processed foods, especially those developed in adolescence, are among the main causes of obesity, which is considered a global public health problem [1–3]. Obesity is a condition that affects nearly 124 million children and adolescents worldwide [4]. Targeted interventions are therefore required to promote dietary and nutrition education in these populations. National dietary recommendations, such as the Dietary Guidelines for Brazilians 2014 [5], have emphasized the importance of monitoring the content of meals, as well as how and when they take place. Meals should be regular and not hurried, and consumed in appropriate locations, in a calm and comfortable environment, and, whenever possible, together with family, friends, or colleagues [5].

Family meals appear to provide several benefits to adolescents, such as the promotion of healthy eating habits [6–9] and a lower risk of overweight [6–10]. Neumark-Sztainer et al. (2010) found a positive impact on food consumption (FC) among youths who had regular family meals (3 or more meals per week), including lower intake of soft drinks and higher intakes of fruits, vegetables, and calcium-rich foods [10]. In another study, Neumark-Sztainer et al. showed that adolescents reporting 7 or more family meals per week had lower intake of snack foods than those reporting fewer family meals [11]. Previous studies have found a significant association between frequency of family meals (FFM) and weigh status, with greater risk of overweight in white youths reporting never eating family meals than in those reporting 3 or more family meals per week, as well as an association of FFM with higher fat mass and body mass index (BMI) z-scores in 17-year-old South African adolescents reporting none or 1 family meal per week [6, 12].

Despite the growing interest in this topic, there is still a dearth of evidence on the effects of regular family meals on FC and nutritional status (NS) in adolescents. Fulkerson et al. (2014) conducted a review to investigate the impact of FFM on dietary and weight outcomes across the lifespan, but only 10 of the selected studies focused on adolescents [7]. Others have also investigated the association between FFM and nutritional outcomes in adolescents, but the reviews had some limitations: one did not consider NS and was limited to articles published in English in 2009 or earlier from only 2 databases [8], and the other also searched only 2 databases, had language restrictions, and included only papers whose main objective was to investigate the relationship between FFM and nutritional outcomes [9]. These observations indicated the need for a systematic review of the evidence for FFM and its association with FC and NS in adolescents.

## Materials and methods

This was a systematic review of studies investigating the association of FFM with NS and/or FC in adolescents aged 10 to 19 years, as defined by the World Health Organization [13]. The review was conducted following the Preferred Reporting Items for Systematic Reviews and Meta-Analyses (PRISMA) guidelines [14], and its protocol was registered with PROSPERO (CRD42017062180; available at https://www.crd.york.ac.uk/PROSPERO/display_record.php?RecordID=62180).

### Data sources, search strategy, and inclusion criteria

No type of meal, publication date, or language restrictions were imposed. Only full-text original articles were included in this review. Quantitative studies of any design were eligible, but qualitative studies were excluded. For the purposes of this review, NS refers to anthropometric measurements and FC refers solely to the intake of food/nutrients and the quality of the diet; thus, studies that associated other aspects of eating behaviors with FFM were not considered. Studies were considered eligible regardless of whether data on FFM was self-reported by the adolescents or reported by parents/legal guardians. Studies were not required to cite the association between these variables as a main objective to be included in the review. Studies involving older children or young adults in addition to adolescents were also considered eligible for inclusion. Investigations of clinical populations or individuals with eating disorders, studies where exposure or outcomes did not occur during adolescence, and studies in which the outcome was not the frequency but rather the quality of family meals were excluded from this review.

**Fig 1:**
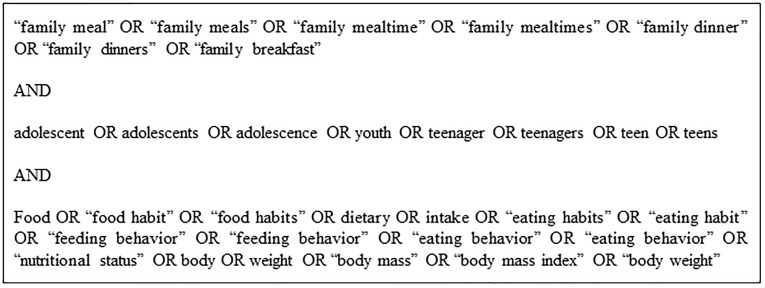
PubMed search strategy.

Studies were identified by searching 5 electronic databases (PubMed, Web of Science, Scopus, BVS Brazil, and Adolec) and gray literature (Google Scholar) and by scanning reference lists of included studies. Google Scholar searches were by relevance and limited to the first 200 citations. Three sets of MeSH terms were used to account for FFM, NS, and FC and needed to be displayed in the title or abstract. The search strategy was initially developed for PubMed (Fig 1) and later adapted for use in all other databases. No filters were applied to any database in the initial search, which was run in August 2019. An updated literature search was performed in May 2020 in all databases using the same search strategy, but with a filter for year of publication (2019–2020).

### Study selection process

Two independent reviewers (R1 and R2) screened titles and abstracts identified by the initial search. Disagreements between the 2 reviewers were resolved by consulting a third reviewer (R3) for arbitration. Duplicates were removed, and the remaining studies were organized using Mendeley Ltd. Full text was obtained for all studies meeting the inclusion criteria and were read independently by 2 reviewers (R1 and R2). Disagreements were resolved by discussion and consensus among R1, R2, and R3, or by consulting an expert (R5). The reference lists of all studies selected for full-text review were hand-searched by R3, and decisions to include additional studies were made in a meeting involving all the aforementioned reviewers. In the update, the titles and abstracts of the articles retrieved were screened by 3 reviewers (R1, R2 and R3) simultaneously, and disagreements were resolved in a discussion meeting. Subsequently, all studies meeting the inclusion criteria were independently read in full by the 3 reviewers (R1, R2 and R3). There were no disagreements at this stage.

### Extraction, risk of bias, and data analysis

The following data were extracted from each study and included in a table designed specifically for this review by 2 of the reviewers (R2 and R3): study characteristics (year of publication, funding sources, and country of origin); sample characteristics (age, recruitment process, and ethnicity); family meal characteristics (type of meal evaluated and FFM); and outcome characteristics (main results, variables measured, and type of assessment). A third reviewer (R1) checked the data for consistency and accuracy.

Risk of bias was assessed by one reviewer (R1), while a second reviewer (R2) reviewed the ratings for consistency and accuracy. Studies were evaluated using the Newcastle-Ottawa scale (NOS) for cohort and cross-sectional studies. The NOS is increasingly used as a measure to assess the quality of nonrandomized trials and provides a separate version for each of these study designs [15, 16]. Published thresholds were used to convert NOS scores into Agency for Healthcare Research and Quality (AHRQ) standards (good, fair, and poor). Because item 4 in the selection domain of the NOS for cohort studies was rated as ‘not applicable’ for all included articles, those with at least 4 stars in other domains were considered of good quality. Investigations with both longitudinal and cross-sectional components were analyzed twice, once for each study design.

Data were compiled into 2 summary tables (NS and FC). A meta-analysis was not possible due to the methodological heterogeneity caused by variations in exposure and outcome measurements across studies, in addition to the absence of confidence intervals and odds ratios in studies that did not investigate the association between FFM and FC or NS as the main objective. A narrative synthesis of extracted findings was conducted by grouping secondary variables into the following 5 categories: dietary habits, lifestyle and social factors, psychological factors, family meal environment, and adolescent health. These findings were discussed by focusing on the association of these variables with FFM, rather than on FFM itself.

## Results

### Search results, design & quality assessment

**Fig 2:**
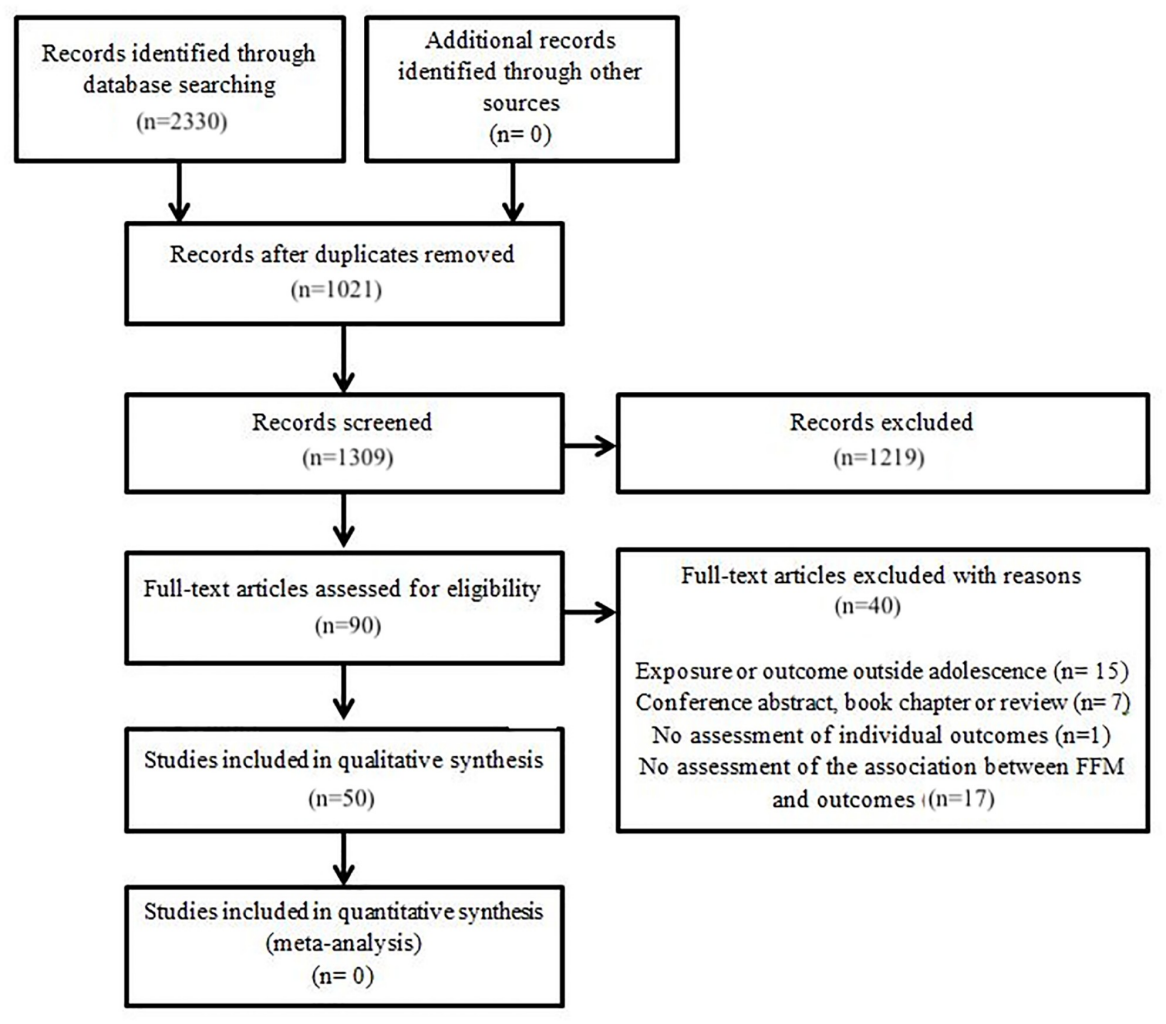
Study selection process.

The initial search yielded 2001 results, of which 47 were included. The search update yielded 329 results, of which 3 were eligible for inclusion, for a total of 50 articles included in this review. The study selection process, including the initial search and the update, is shown in Fig 2. Twenty-five studies investigated the association between FFM and NS [12, 17–40] and are summarized in Table 1. The association between FFM and FC was investigated in 32 studies, which are summarized in Table 2 [11, 17, 22, 23, 27, 30, 38, 39, 41–64]. Seven studies [17, 22, 23, 27, 30, 38, 39] focused on both NS and FC and were included in both tables. Thirty-four were cross-sectional studies [11, 17, 18, 21, 22, 25, 27, 29, 31–35, 37–41, 43–46, 48, 50, 51, 53, 55–62], 12 were longitudinal [19, 20, 24, 26, 28, 29, 31, 42, 47, 49, 63, 64], and 4 studies analyzed both cross-sectional and longitudinal data [12, 36, 52, 54].

**Table 1:** Description of studies investigating the association between family meal frequency and adolescent nutritional status.

Authors	Purpose of the study/Study design	Participants’ characteristics^b^	Definition of FFM	Nutritional status measurements	Relevant findings to family meal & nutritional status
Alamri, 2020 [17]	Purpose: To examine the influence of family meal type (breakfast, lunch, and dinner) on food intake and body mass index (BMI) of Saudi adolescent girls. Design: Cross-sectional	**Sample**: 388 **Age**: 14–16 years old **Country**: Saudi Arabia	“Which meals were consumed with the family?”	Weight and height were measured to calculate BMI percentiles for gender and age	There was a significant negative association between eating family lunch and dinner with adolescent BMI.
Babajafari et al., 2011 [18]	Purpose: To examine the influence of some aspects of family food behaviors on adolescents’ overweight. Design: Cross-sectional	**Sample**: 3862 **Age**: 12–15 years old **Country**: Australia	“How often does your family sit down to eat a meal together?	Weight and height were measured to calculate BMI percentiles and scores standardized for gender and age	Mother’s report on frequency of eating family meal was not significantly associated with overweight adolescents.
Chang & Halgunseth, 2014 [19]	Purpose: To examine the influence of acculturation and ethnicity on the interaction between parental control and family meals on adolescent weight status change. Design: Longitudinal	**Sample**: 6180 **Age**: 11 years old (at fifth grade) **Country**: USA	During the phone interviews, parents reported the number of times in a typical week that ‘‘Your family eats the evening meal together” In addition, parents reported the number of times in a typical week that the family ate breakfast (e.g., ‘‘At least some of the family eats breakfast together”).	Weight and height were measured to calculate BMI percentiles for gender and age	Findings revealed association of high frequency of family meals with unhealthy weight status change for less acculturated Hispanic adolescents who experienced low parental behavioral control at home.
Chen et al., 2019 [20]	Purpose: To examine the associations between multiple aspects of parenting (including parent–child relationship satisfaction concerning love, parental authoritativeness, and family dinner frequency) and various subsequent offspring psychosocial, mental, behavioral, and physical health and well-being outcomes. Design: Longitudinal	**Sample**: 6180 **Age**: 10–17 years old /12.78 years old (mean) **Country**: USA	“How often do you sit down with other members of your family to eat dinner or supper?”	Self-reported weight and height were used to assess nutritional status	Frequency of family dinners was not statistically associated to the nutritional status.
Farajian et al., 2014 [21]	Purpose: To recognize the most important dietary and physical activity habits, sedentary behaviors, plus parental influences that are associated with childhood overweight and Obesity, in a cross-sectional sample of school children. Design: Cross-sectional	**Sample**: 4552 **Age**: 10–12 years old/10.93 years old (mean) **Country**: Greece	All the participants reported the frequency of having meals together with the whole family or at least with one family member.	Weight and height were measured to calculate BMI for gender and age	The frequency of family meals was reported to be a predictor of childhood overweight/obesity.
Frank et al., 2019 [22]	To show current family meal patterns of children and adolescents aged 6 to 17 years living in Germany and investigating associations with sociodemographic characteristics, BMI, and dietary behavior. Design: Cross-sectional	**Sample**: 1355 **Age**: 12–17 years old **Country**: Germany	“In your household, are there certain meals that are always eaten together?”	Self-reported weight and height were used to calculate BMI percentiles for gender and age	Those with overweight eat breakfast, afternoon snacks, or dinner together with their families less frequently than those without overweight.
Fulkerson et al., 2008 [12]	Purpose: To describe associations between the frequency of family meals and overweight status over a 5-year period in a large and ethnically diverse population of adolescent males and females. Design: Cross-sectional and longitudinal	**Sample**: 2516 **Age**: 14.9 years old (mean at baseline) **Country**: USA	“During the past seven days, how many times did all, or most, of your family living in your house eat a meal together?”	Self-reported weight and height to calculate BMI for gender and age	Although significant inverse associations between family meal frequency and overweight status were observed for early adolescent females in all cross-sectional models, longitudinal associations were not significant.
Fulkerson et al., 2009 [23]	Purpose: To examine these associations among a population of adolescents at-risk of academic failure. Design: Cross-sectional	**Sample**: 143 **Age**: 17.2 years old (mean) **Country**: USA	“During the past week, how many days did all, or most of the people you live with eat dinner together?”	Self-reported weight and height to calculate BMI percentiles for gender and age	Adolescents who reported no family dinners in the past week were almost three times more likely to be overweight and six times more likely to be food insecure than adolescents who reported eating 5–7 family dinners per week. Family meal frequency groups did not differ significantly in their report of unhealthy or healthy weight loss practices.
Goldfield et al., 2011 [24]	Purpose: To examine the relationship between the frequency of family meals and BMI in male and female adolescents, while controlling for potential confounding factors associated with BMI, such as parental education, adolescents’ age, and snack-food eating. Design: Longitudinal	**Sample**: 1764 **Age**: 14.12 years old (mean) **Country**: Canada	“Do you eat regular meals with your family at home, sitting at the table together?”	Weight and height were measured to calculate BMI for gender and age	Eating together as a family may be a protective factor against obesity in female adolescents, but not in male adolescents.
Haghighatdoost et al., 2017 [25]	Purpose: To examine the associations of frequency of family dinner with mental disorders and obesity in a nationally-representative sample of Iranian adolescents. Design: Cross-sectional	**Sample**: 5528 **Age**: 10–18 years old; 14.8 (mean for dinner consumers); 14.4 years old (mean for non-dinner consumers) **Country**: Iran	“Typically, how many days per week do you have dinner or supper with your family?” Breakfast and lunch intake were also assessed with a similar question.	Weight and height were measured to calculate BMI for gender and age	In spite of similar dietary intake, family dinner skippers were more likely to be overweight or obese.
Hassan et al., 2019 [26]	Purpose: To determine whether breakfast and family breakfast frequency are associated with adiposity trajectory from early to middle adolescence. Design: Longitudinal	**Sample**: 809 **Age**: 10–16 years old **Country**: Brazil	“How often do you eat the following meals?” followed by a specific item for breakfast frequency in the presence of the father or mother.	Weight and height were measured to calculate BMI for gender and age. Body fat percentage (% BF) was estimated using bioelectrical impedance analysis using a tetrapolar analyzer.	The highest BMI increase within the duration of the study occurred among male participants who did not have breakfast with the family, and the highest decrease in % Body Fat occurred among male participants who had intermediate family breakfast frequency.
Horning et al., 2016 ^a^ [27]	Purpose: To assess correlations between nine parent- and child-reported family dinner frequency measures and evaluate cross-sectional associations between each of the nine family dinner frequency measures and outcomes previously examined with family meal frequency in the research literature. Design: Cross-sectional	**Sample**: 160 **Age**: 8–12 years old/10.3 years old (mean) **Country**: USA	“Did all or most of your family eat dinner together?” “Did you sit down with other people in your family to eat dinner?” “Was at least one parent sitting with you when you ate dinner?”	Weight and height were measured to calculate BMI Z-scores for gender and age	In regard to unadjusted associations between family dinner frequency measures and weight outcomes, all five parent-reported and two of four child-reported dinner frequency measures were significantly and inversely associated with child BMI z-scores.
Horning et al., 2017 ^a^ [28]	Purpose: To examine associations between dinnertime routines and parent dinnertime media use and child and parent BMI outcomes. Also, to examine whether family dinner frequency moderated the relationship between significant mealtime context measures (from aim one) and child and parent BMI outcomes. Design: Longitudinal	**Sample**: 160 **Age**: 8–12.9 years old/10.4 years old (mean) **Country**: USA	“During the past 7 days, how many times were you sitting and eating with your child when he/she ate his/her dinner?”	Weight and height were measured to calculate BMI Z-scores for gender and age	Higher dinnertime routines were significantly associated with lower child BMI z-scores but not parent BMI scores.
Kubik et al., 2009 [29]	Purpose: To determine the prevalence of overweight among a sample of Alternative High School students and assess the association between overweight and selected personal, behavioral, and social environmental factors. Design: Longitudinal	**sample**: 140 **Age**: 14–19 years old/17.3 years old (mean) **Country**: USA	“During the past seven days, how many times did all, or most, of your family living in your house eat dinner together?”	Weight and height were measured to calculate BMI percentiles for gender and age	Overweight students were significantly more likely to report no family meals during the prior week than were normal-weight students.
Larson et al., 2013 ^a^ [30]	Purpose: To examine and compare the frequency of having family meals at breakfast and at dinner according to sociodemographic characteristics. Also, to examine the associations of eating together as a family at breakfast with measures of dietary quality and weight status. Design: Longitudinal	**Sample**: 2793 **Age**: 14.4 years old **Country**: USA	“During the past seven days, how many times did all, or most, of your family living in your house eat breakfast together?”	Weight and height were measured to calculate BMI percentiles for gender and age	Participation in more frequent family breakfast meals was associated with lower risk for overweight/obesity.
Larson et al., 2013 ^a^ [31]	Purpose: To identify the most important home/family, peer, school, and neighborhood environmental characteristics associated with weight status. Design: Cross-sectional	**Sample**: 2793 **Age**: 14.4 years old **Country**: USA	“During the past seven days, how many times did all, or most, of your family living in your house eat a meal together?”	Weight and height were measured to calculate BMI Z-scores for gender and age	Several characteristics of home/family (e.g., infrequent family meals) was consistently associated with higher BMI z-scores among both boys and girls.
Ness et al., 2012 [32]	Purpose: To increase the understanding of risk factors for childhood overweight and obesity among American Indian/Alaska Native (AI/AN) communities. Design: Cross-sectional	**Sample**: 5342 **Age**: 10–17 years old **Country**: USA	“During the past week, how many days did all the family members who live in the household eat a meal together?”	Self-reported weight and height were used to calculate BMI percentiles for gender and age	Family meals were not significantly associated with nutritional status in the sample.
Sedibe et al., 2018 [33]	Purpose: To investigate differences/similarities in dietary habits and eating practices between younger and older, rural and urban South African adolescents in specific environments (home, community, and school) and their associations with overweight and obesity. Design: Cross-sectional	**Sample**: 3490 **Age**: 13 years old (rural); 14 years old (urban) **Country**: South Africa	Participants were asked how frequently they ate their main meal with their family.	Weight and height were measured to calculate BMI Z-scores for gender and age	Eating the main meal with family some days and eating the main meal with family almost every day were associated with increased risk of being overweight and obese among Early Adolescence group.
Sen, 2006 [34]	Purpose: To explore associations between overweight status and the frequency of family dinners (FFD) for adolescents and how those associations differ across race and ethnicity. Design: Cross-sectional	**Sample**: 5014 **Age**: 13.33 years old (mean at baseline) **Country**: USA	It was assessed by asking youth about the number of days that their family ate dinner together in a typical week in the past year.	Self-reported weight and height were used to calculate BMI percentiles for gender and age	For whites, higher FFD was associated with reduced odds of being overweight in 1997, reduced odds of becoming overweight, and increased odds of ceasing to be overweight by 2000.
Smith Price et al., 2009 [35]	Purpose: To examine how several family processes, including parental control, family dinners, parenting style, and demographic variables, are associated with adolescent overweight over time in a large, diverse sample. Design: Cross-sectional	**Sample**: 4688 **Age**: 12–14 years old **Country**: USA	‘‘How many days a week do you eat dinner with your family?”	Weight and height were measured to calculate BMI percentiles for gender and age	More frequent family meals led to decreases in BMI percentile over time.
Taveras et al., 2005 [36]	Purpose: To examine both cross-sectional and longitudinal associations between frequency of family dinner and overweight status in a large sample of 9- to 14-year-old children. Design: Cross-sectional and longitudinal	**Sample**: 14431 **Age**: 9–14 years old **Country**: USA	“How often do you sit down with other members of your family to eat dinner or supper?”	Self-reported weight and height were used to calculate BMI percentiles for gender and age	In cross-sectional analyses, adjusting for potential confounders, the odds of being overweight was 0.85 among children who ate family dinner on “most days” or “every day” compared with those who ate family dinner “never or some days.”
Vik et al., 2016 [37]	Purpose: To assess the prevalence of having regular family breakfast, lunch, dinner among 10–12 years old in Europe, the association between family meals and child weight status, and potential differences in having family meals according to country of residence, gender, ethnicity and parental levels of education. Design: Cross-sectional	**Sample**: 7716 **Age**: 11.5 years old **Country**: Belgium, Greece, Hungary, The Netherlands, Norway, Slovenia, and Spain	“How often do you and/or your spouse/partner have breakfast/lunch/dinner together with your child?”	Weight and height were measured to calculate BMI	Having regular family breakfast, but not lunch or dinner, was inversely associated with overweight.
Utter et al., 2008 [38]	Purpose: To examine associations between frequency of family meals and body mass index (BMI), other aspects of the home food environment, and related nutrition behaviors. Design: Cross-sectional	**Sample**: 3119 **Age**: 14.8 years old (mean) **Country**: New Zealand	"In the last 5 school days, how many times did all or most of your family living in your house eat an evening meal together?"	Weight and height were measured to calculate BMI	Students eating meals with their families on all of the previous five school nights had a lower mean BMI than those who did not eat any meals with their families. When age and gender were treated as confounders in the model, the relationship was no longer significant.
Utter et al., 2013 [39]	Purpose: The aim of the current study is to examine the relationship between family meals and nutrition behaviors of adolescents in New Zealand. Design: Cross-sectional	**Sample**: 8734 **Age**: 13–17 years old **Country**: New Zealand	"During the past 7 days, how many times did all, or most, of your family living in your house eat a meal together?"	Weight and height were measured to calculate BMI	There were no relationships between frequency of family meals and BMI. The mean BMI of students sharing meals 7 or more times a week (22.6) was similar to that of those students sharing family meals twice a week or less often (23.2) after controlling for the demographic characteristics of students.
Wurbach et al., 2009 [40]	Purpose: To describe the meal patterns of Jena schoolchildren and their associations with children’s weight status and parental characteristics. Design: Cross-sectional	**Sample**: 1571 **Age**: 7–14 years old **Country**: Germany	Variables used to describe the Family meals of the children were the following: The frequency of main meals (breakfast, lunch, dinner) eaten together with all family members.	Weight and height were measured to calculate BMI Z-scores for gender and age	Using linear regression analysis, a high inverse association between BMI standardized scores and meal frequency was observed.

^a^ Same studies with each outcome explored in a different published article.

^b^ Information about participants’ age was reported according to the data available in the studies.

BMI: body mass index

**Table 2:** Description of studies investigating the association between family meal frequency and adolescent food consumption.

Authors	Purpose of the study	Participants’ characteristics b	Definition of FFM	Food consumption measurement tools	Relevant findings to family meal & Food consumption
Alamri, 2020 [17]	Purpose: To examine the influence of family meal type (breakfast, lunch, and dinner) on food intake and body mass index (BMI) of Saudi adolescent girls. Design: Cross-sectional	**Sample**: 388 **Age**: 14–16 years old **Country**: Saudi Arabia	“Which meals were consumed with the family?”	Three-day dietary records	Eating a family breakfast was positively associated with adolescent intake of dairy products and meat alternatives. Eating a family lunch was positively associated with adolescent intake of vegetables and meat alternatives. And Eating a family dinner was positively associated with adolescent intake of fruit, vegetables, dairy products, and whole grains
Arcan et al., 2019 [41]	Purpose: To examine the prevalence of parental report of children’s adherence to USDA’s MyPlate guidelines of ‘half of plate filled with fruits and vegetables (FV)” and to examine what food related practices were associated with frequency of serving half the plate of FV. Design: Cross-sectional	**Sample**: 160 **Age**: 8–12 years old **Country**: USA	“During the past seven days, how many times did all or most of your family living in your home eat 'dinner together?”	-Three 24-hour dietary recall interviews -Healthy Eating Index (HEI)-2010 -Home Food Inventory	Significant positive associations were observed between the "half plate FV" and Family dinner frequency.
Burgess-Champoux et al., 2009 [42]	Purpose: To examine the longitudinal associations of participation in regular family meals with eating habits during adolescence. Design: Longitudinal	**Sample**: 677 **Age**: 12.8 years old (mean at baseline); 17.2 years old (mean at follow-up) **Country**: USA	‘‘During the past 7 days, how many times did all, or most, of your family living in your house eat a meal together?”	-Youth and adolescent FFQ (YAQ) -The Project EAT survey instrument	Family meals played an important role in enhancing overall diet quality during the transition from early to middle adolescence. The more the adolescents participated in family meals, the more healthful was its diet. Regular family meals were positively associated with mean daily intakes of vegetables, calcium-rich food, dietary fiber, and several nutrients, and among males, regular family meals were negatively associated with fast-food intake on longitudinal results.
Conlon et al., 2019; Conlon et al., 2015 [43]	Purpose: To evaluate cross-sectional associations between children’s diet and physical activity behaviors and components of the home environment, including parenting practices, family meal habits, home availability of fruits/ vegetables, home availability of sugar-sweetened beverages, and home availability of screen-media and physical activity resource. Design: Cross-sectional	**Sample**: 677 **Age**: 12.8 years old (mean at baseline); 17.2 years old (mean at follow-up) **Country**: USA	“How many times does your family sit down together for dinner?”	-The Block Kids 2004 FFQ	Children that had more frequent family meals were more likely to consume fruits and vegetables.
Demissie et al., 2015 [44]	Purpose: To examine behavioral and environmental factors that may be related to dietary behaviors among U.S. high school students. Design: Cross-sectional	**Sample**: 11,429 **Age**: not reported (high school students) **Country**: USA	“During the past 7 days, on how many days did you eat dinner or an evening meal with a parent or guardian?”	-Nutrition Study questionnaire	For both genders, eating dinner with a parent or guardian 5 to 7 days during the past week was associated with higher odds of consuming at least three healthful foods or beverages
Feldman et al., 2007 [45]	Purpose: To examine associations between watching television during family meals and dietary intake among adolescents. Design: Cross-sectional	**Sample**: 4746 **Age**: 11–18 years old/14.9 years old (mean) **Country**: USA	“During the past seven days, how many times did all, or most, of your family living in your house eat a meal together?”	-The Project EAT survey instrument -149-item Youth and adolescent questionnaire	Adolescents watching TV during family meals are more likely to have a poorer quality diet compared to adolescents eating family meals without watching TV. Watching TV during family meals was associated with lower intakes of vegetables, grains, and dairy food, and higher intakes of soft drinks and fried food.
Fink et al., 2014 [46]	Purpose: To examine the relationship between diet quality and frequency of family meals throughout childhood and adolescence. Design: Cross-sectional	**Sample**: 824 **Age**: 12–17 years old **Country**: USA	“How many times in a typical week do members of your household eat a main meal together that was prepared at home?”	-2010 North Carolina Child Health Assessment and Monitoring Program survey	In adjusted analyses, participating in 5 or more family meals/week was associated with greater vegetable intake and greater fruit intake among participants from 12–17 years old.
Frank et al., 2019 [22]	To show current family meal patterns of children and adolescents aged 6 to 17 years living in Germany and investigating associations with sociodemographic characteristics, BMI, and dietary behavior. Design: Cross-sectional	**Sample**: 1355 **Age**: 12–17 years old **Country**: Germany	“In your household, are there certain meals that are always eaten together?”	-FFQ and Computer-assisted diet history interview (DISHES)	Daily consumption of sugary drinks is lower in 12 to 17-year-old adolescents who have breakfast with their families every day/often than in adolescents who rarely/never have breakfast with their family. Significant associations were not found for fruit, vegetables, water, Confectionery and salty snacks, milk products, meat sausages, cereal products, fast food, and fishes.
Fulkerson et al., 2009 [23]	Purpose: To examine the associations between family dinner frequency and dietary practices and psychosocial well-being, and inverse associations between family dinner frequency and overweight status among a population of adolescents at-risk of academic failure. Design: Cross-sectional	**Sample**: 143 **Age**: 17.2 years old (mean) **Country**: USA	“During the past week, how many days did all, or most of the people you live with eat dinner together?”	-Trained research staff administered the survey questionnaire	Family dinner frequency was significantly positively associated with daily fruit consumption. Adolescents reporting 5–7 family dinners per week had a significantly higher daily servings of fruit consumption than adolescents reporting fewer family dinners.
Gillman et al., 2000 [47]	Purpose: To examine the associations between frequency of eating family dinner and several measures of diet quality in a large national sample of 9- to 14 years-old children. Design: Longitudinal	**Sample**: 16202 **Age**: 9–14 years old **Country**: USA	“How often do you sit down with other members of your family to eat dinner or supper?”	-Validated semi-quantitative FFQ	Eating family dinner was associated with healthful dietary intake patterns; including more fruits and vegetables, less fried food and soda, less saturated, and trans-fat, lower glycemic load, more fiber and micronutrients from food, and no material differences in red meat or snack foods.
Giray & Ferguson, 2018 [48]	Purpose: To explore the quantity (frequency) and quality (priority, atmosphere, structure) of family mealtimes and associations with nutritional and emotional health in Jamaica. Design: Cross-sectional	**Sample**: 330 **Age**: not reported (grades 7–8 and 10–11) **Country**: Jamaica	Participants reported the “number of times family had a meal together in the past week”	-Family Mealtimes Questionnaire -Jamaican Youth Risk and Resiliency Behavior Survey (Adapted)	The frequency of family meals was not associated with diet quality.
Granner & Evans, 2011 [49]	Purpose: To assess individual, social, and family environmental factors related to fruit and vegetable intake among white and black adolescents aged 11–15 years old. Design: Longitudinal	**Sample**: 736 **Age**: 11–15 years old **Country**: USA	The Block Food Frequency Questionnaire Short Form was used to measure the frequency of family meals.	-Block FFQ (short form)	Family dinner frequency, was significantly associated with fruit and vegetable intake only when comparing the <3 servings per day with 3–4 servings per day category and when comparing the <3 servings per day with the >5 servings per day category.
Haapalahti et al., 2003 [50]	Purpose: To describe the meal patterns and food use on weekdays among 10- to 11-year-old Finnish children and to analyze these in relation to family’s socioeconomic status and the child’s behavior. Design: Cross-sectional	**Sample**: 404 **Age**: 10–11 years old **Country**: Finland	The main family meals were assessed using the following statements: ‘We tend to eat at the same time’, ‘The whole family tends to eat together’ and ‘We prepare a hot meal’.	- FFQ	Participants with no regular family dinner ate sweets and fast foods more often than those with regular family dinner.
Hong et al., 2019 [51]	Purpose: To assess the nutritional quality of breakfast among Korean school-aged children and adolescents depending on eating together as a family, based on the 2013–2014 Korea National Health and Nutrition Survey. Design: Cross-sectional	**Sample**: 1831 **Age**: 6–17 years old/11 years old (mean) **Country**: South Korea	“When you had breakfast in the last year, did you usually eat with others?”	-EAT 2010 survey -Semi-quantitative Youth and Adolescent FFQ	Food intake and intake of carbohydrates and iron were higher in the family breakfast group The average numbers of serving for “Grains” and “Vegetables” food groups and the average Dietary Diversity Score were significantly higher in family breakfast group. Prevalence of consumption of milk and dairy products was higher in the breakfast group alone.
Horning et al., 2016 [27]	Purpose: To assess correlations between nine parent- and child-reported family dinner frequency measures and evaluate cross-sectional associations between each of the nine family dinner frequency measures and outcomes previously examined with family meal frequency in the research literature. Design: Cross-sectional	**Sample**: 160 **Age**: 8–12 years old/10.3 years old (mean) **Country**: USA	“Did all or most of your family eat dinner together?” “Did you sit down with other people in your family to eat dinner?” “Was at least one parent sitting with you when you ate dinner?”	-Three 24-hour dietary recalls -The Healthy Eating Index-2010	Both parent- and child-reported family dinner frequency were significantly associated with children’s average daily servings of fruits and vegetables and dietary quality.
Larson et al., 2007 [52]	Purpose: To examine if family meal frequency during adolescence predicts more structured eating, priority for social eating, and better dietary intake during the transition to adulthood. Design: Cross-sectional and longitudinal	**Sample**: 1710 **Age**: mean—15.9 years old (time 1); 20.4 years old (time 2) **Country**: USA	“During the past seven days, how many times did all, or most, of your family living in your house eat breakfast together?”	-EAT 2010 survey -Semi-Quantitative Youth and Adolescent FFQ	Family meal patterns during adolescence predicted diet quality during early young adulthood.
Larson et al., 2013 ^a^ [30]	Purpose: To examine and compare the frequency of having family meals at breakfast and at dinner according to sociodemographic characteristics and to examine the associations of eating together as a family at breakfast with measures of dietary quality and weight status. Design: Longitudinal	**Sample**: 2793 **Age**: 14.4 years old (mean) **Country**: USA	“During the past seven days, how many times did all, or most, of your family living in your house eat breakfast together?”	-EAT 2010 survey Semi-Quantitative Youth and Adolescent FFQ	Participation in more frequent family breakfast meals was associated with several markers of better diet quality.
Larson et al., 2016 [53]	Purpose: To describe the frequency of having family meals at breakfast in relation to the frequency of having family meals at dinner along with patterns of purchasing family breakfast meals at fast-food restaurants and how family breakfast meals are served. Design: Cross-sectional	**Sample**: 827 **Age**: 14–16 years old **Country**: USA	“During the past seven days, how many times did all, or most, of your family living in your house eat a meal together?”	-Project BreakFAST survey -Three 24-hour dietary recalls	Frequency of eating breakfast together was unrelated to most markers of diet quality among adolescent boys and girls who report skipping breakfast on two or more days per week. Differences in scores by frequency of eating breakfast together were identified only for whole fruit among boys and refined grains and empty calories among girls. Adolescent boys who ate breakfast together with their family one to two times in the past week had poorer scores for whole fruit than their peers who never ate breakfast with their family. For adolescent girls, frequency of eating breakfast together was associated with poorer scores for refined grains but with better scores for empty calories (i.e., consumed fewer calories from solid fats, alcohol, and added sugars).
Leech et al., 2014 [54]	Purpose: To examine cross-sectional and longitudinal associations between food involvement, frequency of family dinner meals and dietary patterns among children aged 10–12 years old. Design: Cross-sectional and longitudinal	**Sample**: 155 **Age**: 10–12 years old/11.5 years old (mean) **Country**: Australia	Frequency of family meals was measured asking parents how often their child ate dinner with them at home.	-FFQ whose items were identified from the 1995 Australian National Nutrition Survey	Cross-sectionally, daily dinner meals with family were positively associated with a healthful dietary pattern and lower scores of energy-dense pattern for boys.
Lipsky et al., 2015 [55]	Purpose: To examine trends and changes in eating behaviors during the adolescent-adult transition in a contemporary, nationally representative U.S. cohort, and to examine whether these trends differ by sociodemographic factors or baseline weight status. Design: Longitudinal	**Sample**: 2785 **Age**: 16–20 years old/16.27 years old (mean) **Country**: USA	“How often do you have an evening meal together with your mother/stepmother or father/stepfather?”	-Questionnaire based on Youth Risk Behavior Surveillance System and the multinational Health Behavior in School-Aged Children study	Fruit/vegetable intake frequency was associated positively with family meals and breakfast, and inversely with fast food, while whole grain intake frequency was associated positively with family meals.
Lipsky et al., 2017 [56]	Purpose: To examine behavioral correlates and baseline predictors of diet quality over the transition to adulthood in a contemporary, diverse national cohort of US 10th graders. Design: Longitudinal	**Sample**: 566 **Age**: 16.5 years old (mean) **Country**: USA	“How often do you have an evening meal together with your mother/stepmother or father/stepfather?”	-Three non-consecutive 24-hour dietary recalls -Healthy Eating Index-2010	Better diet quality was associated with greater family meals.
Makansi et al., 2019 [57]	Purpose: To describe eating behaviors of adolescents in Dubai and the factors associated with fruit and vegetable intake. Design: Cross-sectional	**Sample**: 620 **Age**: 15–18 years old **Country**: United Arab Emirates	“During the past seven days, how many times did all or most of your family living in your home eat dinner together?”	-EAT survey Youth Risk Behavior Surveillance Survey	Family meals were not significantly associated with daily intake of fruits and vegetables.
Martins et al., 2019 [58]	Purpose: To investigate how often Brazilian adolescents eat meals with their parents and verify the association between this habit and quality of the diet. Design: Cross-sectional	**Sample**: 102072 **Age**: 11–19 years old **Country**: Brazil	“Do you usually eat lunch or dinner with your mother, father, or guardian?”	-Smartphone questionnaire	Eating meals with parents at least 5 days a week was positively associated with frequent consumption of beans, and vegetables and negatively associated with frequent consumption of sweets, ultra-processed salty foods, and fried salty snacks.
Neumark-Sztainer et al., 2003 [11]	Purpose: To examine family meal patterns and associations with sociodemographic characteristics and dietary intake in adolescents. Design: Cross-Sectional	**Sample**: 4629 **Age**: 14.9 years old (mean) **Country**: USA	“During the past seven days, how many times did all, or most, of your family living in your house eat a meal together?”	-Youth and Adolescent FFQ	Frequency of family meals was positively associated with the intake of fruits, vegetables, grains, and calcium-rich foods, and negatively associated with soft drink intake. Youths reporting at least seven family meals had lower intakes of snack foods than youths reporting fewer family meals.
Oliveira et al., 2018 [59]	Purpose: To investigate the association of dietary intake with eating behavior, screen time, and physical activity among Brazilian adolescent students. Design: Cross-sectional	**Sample**: 14653 **Age**: 14.0 years old (mean) **Country**: Brazil	“How often do you have lunch or dinner with your parents during the week?”	-Online questionnaire	The adolescents who had had lunch or dinner with their parents or who had frequent meals at home (five or more days a week) had a healthier diet, with higher mean days of consumption of beans, vegetables, cooked vegetables, and milk. Conversely, they had less frequent consumption of French fries, fried snacks, cold cuts, sweets, and soft drinks during the week.
Totland et al., 2017 [60]	Purpose: To describe family meal patterns among 11-year-old children across Europe and identify correlates of irregular family breakfast and dinner consumption in different regions of Europe. Design: Cross-sectional	**Sample**: 13305 **Age**: 9–14 years old/11.3 years old (mean) **Country**: Austria, Belgium, Denmark, Iceland, the Netherlands, Norway, Portugal, Spain, and Sweden	“How often do you have breakfast/dinner (supper/evening meal) with your mother and/or father?”	-Questionnaire elaborated to the Pro Children project	Correlates of irregular family breakfasts and dinners were less vegetable consumption. Irregular family breakfasts were associated with more television viewing.
Utter et al., 2008 [38]	Purpose: To examine associations between frequency of family meals and body mass index (BMI), other aspects of the home food environment, and related nutrition behaviors. Design: Cross-sectional	**Sample**: 3119 **Age**: 14.8 years old (mean) **Country**: New Zealand	"In the last 5 school days, how many times did all or most of your family living in your house eat an evening meal together?"	-Youth’07 Survey	Frequency of family meals was associated with consuming five fruits and vegetables a day, eating breakfast, and bringing a lunch from home.
Utter et al., 2013 [39]	Purpose: The aim of the current study is to examine the relationship between family meals and nutrition behaviors of adolescents in New Zealand. Design: Cross-sectional	**Sample**: 8734 **Age**: 13–17 years old **Country**: New Zealand	"During the past 7 days, how many times did all, or most, of your family living in your house eat a meal together?"	-Youth’07 Survey	Frequency of family meals were associated with greater consumption of fruits and vegetables and breakfast. Adolescents who frequently shared family meals were also more likely to report that what they ate in the past week was healthier than adolescents who did not.
Walton et al., 2018 [61]	Purpose: To examine whether level of family functioning is associated cross-sectionally with frequency of family dinners and dietary intake among a US national sample of adolescents and young adults. Design: Cross-sectional	**Sample**: 2728 **Age**: 19.4 years old (mean) **Country**: USA	“How often do you sit down with other members of your family to eat dinner or supper?”	-27-item FFQ for Fruit and Vegetable intake	More frequent family dinners were associated with improved dietary intake. Among female participants, family dinners were associated with higher intakes of fruits and vegetables and lower consumption of fast food and takeout food in models adjusted for age, mothers’ spouse or partner’s educational attainment, and family structure. Among male family members, participation in more frequent family dinners was significantly associated with higher intakes of fruits and vegetables and lower consumption of fast food, takeout food, and sugar-sweetened beverages, when adjusted for age, mothers’ spouse or partner’s educational attainment, and family structure.
Woodruff et al., 2009 [62]	Purpose: To examine associations between family dinner frequency and fast food frequency, soft drink consumption, breakfast skipping, dieting for weight loss, concerns about a high body weight, and self-efficacy for healthy eating during certain situations. Design: Cross-sectional	**Sample**: 3223 **Age**: 10–14 years old **Country**: Canada	‘‘Typically, how many days per week do you eat dinner or supper with at least one parent?”	-24-hours diet recall -Waterloo Web-based Eating Behavior Questionnaire	Higher family dinner frequency was significantly associated with less soft drink consumption, consuming breakfast on the day of the survey, having higher self-efficacy for healthy eating when at home with family, and during social times with friends.
Woodruff et al., 2010 [63]	Purpose: To describe family dinner frequency (FDF) and its associations with overall diet quality. Design: Longitudinal	**Sample**: 1293 **Age**: 10–14 years old **Country**: Canada	‘‘Typically, how many days per week do you eat dinner or supper with at least one parent?”	-24-hours diet recall -US-based Healthy Eating Index -Waterloo Web-based Eating Behavior Questionnaire	Diet quality scores were higher among participants reporting 6–7 dinners/week.
Woodruff et al., 2014 [64]	Purpose: To determine the associations between the frequency and calorie consumption of meals/snacks and family meals. Design: Longitudinal	**Sample**: 964 **Age**: 10–14 years old; **Country**: Canada	"Typically, how many days per week do you eat dinner/supper with at least one parent/guardian?”	-24-hours diet recall -Waterloo Web-based Eating Behavior Questionnaire	Specifically, for the dinner meal, fewer calories were consumed if the dinner meal was consumed with family members compared with eating dinner with friends.

^a^ Same studies with each outcome explored in a different published article.

^b^ Information about participants’ age was reported according to the data available in the studies.

FFQ: Food Frequency Questionnaire

The cross-sectional and longitudinal components of studies with both types of data were evaluated separately for risk of bias. According to the NOS, 35 studies were rated as having good quality (27 cross-sectional and 8 longitudinal) [12, 17–26, 32–38, 42–45, 47, 48, 50–52, 54, 56, 58–61, 63, 64], whereas 19 were of fair quality (15 cross-sectional and 4 longitudinal) [11, 20, 27–31, 36, 39–41, 46, 49, 52–55, 57, 62]. In most cases, cross-sectional studies were rated as having fair quality because of lack of justification of sample size, no calculation of the response rate, or no description of characteristics of responders and non-responders, and longitudinal studies because of written self-report questionnaires to investigate exposure, follow-up rate <80%, or no description of participants lost to follow-up.

### Overview of included studies

The studies included in this review were published between 2000 and 2020, with 23 published in the past 5 years [17, 22, 25–29, 33, 37, 41, 43, 44, 48, 49, 51, 53, 55–61]; of these, 8 were published between 2019 and 2020 [17, 20, 22, 26, 41, 43, 51, 58]. Thirty-one studies were conducted in North America [11, 12, 19, 20, 23, 24, 27–32, 34, 35, 36, 41–47, 49, 52, 53, 55, 56, 61–64], and 27 of these were conducted in the United States. An additional 6 studies were performed in Europe [21, 22, 38, 41, 50, 60], 4 in Oceania [18, 39, 40, 54], and 4 in Latin America [26, 48, 58, 59]. Three studies were performed in Asia [25, 51, 57], 1 in Africa [33], and 1 in the Middle East [17].

#### Population

Sample sized ranged from 140 [29] to 102 072 [58] participants, with 32 studies assessing over 1000 individuals [11, 12, 18–22, 24, 25, 30–40, 44, 45, 47, 51, 52, 55, 58–63]. Except for 1 study that evaluated only females [17], all others included both males and females. Thirteen studies provided no information on participant ethnicity [21, 22, 25, 26, 40, 49–51, 54, 59, 60, 62, 63]. The other studies described ethnicity and/or race in different ways, but in most of them (n = 24) samples were predominantly Caucasian or non-Hispanic white [11, 12, 18–20, 27–29, 32, 34–36, 41, 44–47, 49, 52, 53, 55, 56, 61, 64].

#### Assessment of FFM

Each study assessed the frequency of at least one main meal (breakfast, lunch, dinner/supper/evening meal). Of 50 studies, 17 assessed the frequency of meals in general [11, 12, 18, 21–24, 32, 35, 39, 42, 45, 46, 48, 49, 53, 57], 11 assessed dinner, supper, or evening meals [20, 25, 36, 38, 47, 55, 56, 61–64], 8 assessed dinner only [27–29, 34, 41, 43, 50, 54], 5 assessed all 3 main meals (breakfast, lunch, and dinner) [17, 33, 37, 40, 44], 4 assessed only breakfast [26, 31, 51, 52], 3 assessed breakfast and dinner or evening meals [19, 30, 60], and 2 assessed lunch and dinner [58, 59].

Most studies (n = 40) analyzed FFM over a 1-week period [11, 12, 18–25, 27, 28, 30–35, 37, 39, 41, 42, 44–46, 48–50, 52–64]. However, 5 studies did not provide a specific time frame [20, 26, 29, 36, 43], while 2 studies assessed meals in the last 5 weekdays [38, 40], 1 in the last 3 days [17], 1 in a single day [47], and 1 focused on usual meals during the past year [51].

FFM was self-reported by the adolescents in 34 studies [11, 12, 17, 20, 21, 23–26, 29, 30, 33–36, 38, 39, 42, 44, 45, 47, 49, 51–53, 55–60, 62–64] and reported by parents in 8 studies [22, 32, 37, 40, 41, 43, 46, 54]. In 7 studies, these data were reported by both parents and adolescents [18, 19, 27, 28, 31, 48, 50]. One study did not describe how these data were collected [61].

Most studies (n = 25) investigated FFM using multiple-choice questions, with responses limited to the time period under consideration (e.g., never, 1–3 times/week, 4–6 times/week, every day) or overall frequency (e.g., regularly/sometimes/never) [11, 12, 17, 18, 20–24, 26, 30, 33, 34, 36, 38–40, 47, 48, 52, 53, 57, 59, 62–64]. Seven studies calculated the mean number of family meals in the period investigated [19, 27, 28, 35, 42, 55, 56], and 9 studies analyzed this variable divided into 2 categories (e.g., less than 5 or at least 5 meals a week; regularly or irregularly) [29, 32, 43, 46, 50, 51, 58, 60, 61]. Three studies also described the percentage of participants reporting different FFMs in the period under consideration (e.g., 3 or more family meals in the previous week; 5 to 7 meals in the previous week) [37, 44, 45]. Five studies did not provide descriptive data on FFM [25, 31, 41, 49, 54], and 1 study reported FFM as the number of meals in a day [17].

#### Other outcome variables

In addition to FC and/or NS, 30 studies also evaluated lifestyle and social factors [18–21, 24, 26, 29, 30–32, 35–41, 43–45, 50–53, 55, 56, 59–62], such as physical activity, screen time, family structure, peer characteristics, household food insecurity, and availability of specific foods in the home. Twenty-seven studies also investigated dietary practices [21, 23, 24, 26, 33, 36, 38–42, 44–49, 51–54, 56–58, 62–64], such as dieting, purchasing breakfast, snacking patterns, and eating behaviors. Twelve studies also evaluated family meal environment [21, 23, 24, 27, 28, 41, 43, 48, 51, 53, 54, 56], including variables such as mealtime conversation, family dinnertime routine, importance of mealtime, watching television during meals, parenting style, and parent dinnertime media use. Five studies assessed psychological factors [18, 20, 25, 48, 50], such as behavior problems and mental health. Five studies evaluated adolescent health factors [17, 20, 23, 32, 36], such as Tanner stages, physical health, health condition, and beginning of menstrual life for girls.

### Main outcomes of FFM & NS

Ten studies investigated the association between FFM and BMI classifications. Of these, 6 identified a positive relationship between FFM and better NS. Alamri (2020) examined the influence of family meal type (breakfast, lunch, and dinner) on food intake and BMI of Saudi adolescent girls and found that eating a family lunch or dinner was negatively associated with adolescent BMI; however, no significant association was found between family breakfast and adolescent BMI [17]. Chang & Halgunseth (2014) examined the influence of acculturation, ethnicity, interactions among family meals, parental control, and FFM on changes in NS in Hispanic and Caucasian adolescents in the United States over approximately 4 years. The results showed that non-Hispanic white adolescents were more likely to maintain a healthy weight, not to purchase school lunch, and to eat more family meals. There was also an association between high FFM and a negative change in NS (normal to overweight) in Hispanic adolescents with lower parental behavioral control (high behavioral control and use of power assertive parenting techniques to address child misbehavior are part of the demandingness dimension of parenting style framework) [19]. Farajian et al. (2014) identified FFM as 1 of the 5 main predictors of overweight in childhood, as having frequent family meals was associated with a reduced risk of overweight and obesity [21]. Larson et al. (2013) found that a higher frequency of family breakfasts was associated with a lower risk of overweight [30]. Kubik et al. (2009) showed that obese students were more likely than their normal-weight peers to report no family meals in the previous week [29]. Sen (2006) assessed FFM and NS in individuals of different ethnicities and found that, for white adolescents, high FFM was associated with reduced weight in 1997 and with a reduced likelihood of becoming overweight and increased odds of ceasing to be overweight in 2000 [34]. Vik et al. (2016) evaluated the association between eating the 3 main meals with family members and NS in adolescents and showed that having regular family breakfast, but not lunch or dinner, was inversely associated with overweight [37]. The other 3 studies investigating these variables did not identify any significant association between them [20, 32, 40].

Eight studies evaluated the association between FFM and NS as defined by BMI. Six of these studies identified a negative association between exposure and outcome. Fulkerson et al. (2008) evaluated NS and FFM in adolescents over the course of 5 years and reported that, although these variables were not longitudinally associated, significant inverse relationships occurred between FFM and overweight in female adolescents in all cross-sectional models [12]. Goldfield et al. (2019) found that higher FFM was associated with lower BMI only in female participants [24]. Haghighatdoost et al. (2017) observed that family dinner frequency was inversely related to obesity in adolescents [25]. Hassan et al. (2019), in a 3-year longitudinal study, showed that male participants who did not have breakfast with their families had the largest increases in BMI over the course of the study, while the largest reductions in percent body fat were observed in male adolescents who reported an intermediate frequency of family breakfasts [26]. Taveras et al. (2005), in cross-sectional analyses, identified a lower likelihood of becoming overweight in participants who ate family dinner on “most days” or “every day” than in those who ate family dinner “some days or never”; no significant associations were observed in longitudinal analyses [36]. In the studies conducted by Babajafari et al. (2011) [18], Utter et al. (2008) [38], and Utter et al. (2013) [39], no significant associations were observed between FFM and NS in adolescents.

Seven studies also evaluated the association between FFM and NS using BMI z-scores or age-specific percentiles, and all of them identified positive associations between FFM and lower odds or prevalence of overweight. Frank et al. (2019), aiming to show current family meal patterns of children and adolescents aged 6 to 17 years living in Germany and to investigate associations with sociodemographic characteristics, BMI, and dietary behavior, found that overweight participants aged 12–17 years ate breakfast, afternoon snacks, or dinner together with their families less frequently than their non-overweight peers [22]. Fulkerson et al. (2009) reported that adolescents who did not eat dinner with their families in the week before the study were almost 3 times more likely to be overweight [23]. Horning et al. (2016), evaluating different measures of FFM, as reported by parents and adolescents, and the relationship between these variables and BMI z-scores, found, in adjusted analyses, that measures of sitting and eating together were inversely related to BMI z-scores in adolescents [27] In another study, Horning et al. (2017) evaluated the moderating role of FFM in the relationship between meal context and NS of parents and adolescents, and the results suggested that FFM amplifies the relationship between healthy eating behavior and BMI in the context of family meals [28]. Larson et al. (2013) showed that infrequent family meals were consistently associated with higher BMI z-scores in both boys and girls [31]. Sedibe et al. (2018), in cross-sectional analyses, showed that eating the main meal with the family on “some days” or “nearly every day” was 1 of the 2 main variables responsible for an increased risk of overweight and obesity only in black or rural preadolescents [33]. The only study to use BMI percentiles was conducted by Smith Price et al. (2009), who found that more frequent family meals led to improvements in NS over time [35].

### Main outcomes of FFM & FC

Most studies (n = 24) used food-frequency questionnaires that had been previously validated or developed specifically for the study [11, 22, 23, 30, 38, 39, 42–52, 54, 55, 57–61]. The remaining studies used 24-h dietary records [17, 27, 41, 53, 56, 62–64].

Using a food-frequency questionnaire, Fulkerson et al. (2009) analyzed breakfast content, dietary habits, fruit and vegetable consumption, and unhealthy food intake and found that family dinner frequency was positively associated with breakfast frequency and daily fruit intake, but not with fast-food restaurant use, regular soft drink consumption, vegetable consumption, combined fruit and vegetable consumption, or high-fat food intake [23]. Frank et al. (2019), in a subsample from the German Health Interview and Examination Survey for Children and Adolescents (KiGGS/2014-2017), investigated an association between FFM and food intake or the frequency of consumption of food groups and found that the daily consumption of sugary drinks is lower in adolescents aged 12–17 years who have breakfast with their families every day/often than in those who rarely/never have breakfast with the family. Analyses for other food groups, including fruit, vegetables, water, confectionery/salty snacks, milk products, meat/sausages, cereal products, fast food, and fish, showed no significant associations [22]. Larson et al. (2013) evaluated the association between frequency of family breakfasts and food intake and found that adolescents who ate more meals with their families were more likely to have positive dietary habits, including increased consumption of fruit, whole grains, fiber, and potassium, as well as reduced consumption of sugar-sweetened beverages [30]. In the studies conducted by Utter et al. (2008) and Utter et al. (2013), a positive association was identified between higher fruit and vegetable intake and frequency of family breakfast [38, 39].

Burgess-Champoux et al. (2009) examined the longitudinal association between participation in family meals and dietary habits in adolescence over a 5-year period. Male and female adolescents who ate regular family meals (at least 5 per week) had higher intakes of vegetables, calcium-rich foods, fiber, and several vitamins and minerals [42]. Conlon et al. (2019) assessed the influence of family environment on obesity-related health behaviors in adolescents and showed that participants with higher FFM were more likely to eat fruits, but they did not differ from their peers in the consumption of vegetables or sugar-sweetened beverages. Additionally, adolescents whose parents reported a higher frequency of television watching during family meals were likely to eat less fruit [43]. Demissie et al. (2015) classified participants’ dietary habits as healthy or unhealthy based on the intake of different food groups and found that healthier eating habits were associated with higher FFM [44].

Feldman et al. (2007) reported that adolescents who watched television during family meals were more likely to have a poorer diet than those who did not. Television watching during family meals was associated with lower intakes of vegetables, grains, and dairy products and with higher intakes of soft drinks and fried foods. Additionally, the results showed that adolescents eating regular family meals while watching television had better dietary intake than those not eating regular family meals [45]. Fink et al. (2014) also found that having 5 or more family meals per week was associated with higher intakes of vegetables and fruit in participants aged 12–17 years [46]. Gillman et al. (2000) reported that a higher frequency of family dinners was associated with healthier eating habits, including higher intakes of fruit, vegetables, fiber, and micronutrients and lower intakes of fried foods, soft drinks, and saturated and trans fats. In addition, FFM was associated with lower glucose levels, although it was not significantly associated with the intake of red meat or snack foods [47]. Hong et al. (2019), using data from a national Korean survey to compare adolescents who had breakfast with their families with those who had this meal alone, performed analyses for the association between FFM and nutritional quality, food intake, meal frequency, and energy intake and found that the food intake of carbohydrates and iron, the average Dietary Diversity Score, and the number of servings of grains and vegetables were significantly higher in the family breakfast group. However, the consumption of milk and dairy products was higher in the group of participants eating breakfast alone [51].

Giray & Ferguson (2018), examining the association between the frequency and quality of family meals and nutritional outcomes in young Jamaicans, found no significant associations between these variables [48]. Granner & Evans (2011), evaluating individual characteristics, social factors, family environment and their relationship to fruit and vegetable intake in adolescents, showed, in multivariate analyses, a positive association between the frequency of family dinners and daily fruit and vegetable intake, as evidenced by differences between adolescents who ate fewer than 3 portions and those who ate 3–4 portions of fruit and vegetables per day, and between those who ate fewer than 3 portions and those who ate more than 5 portions per day [49]. Haapalahti el at. (2003) found a significant association between FFM and healthier eating habits, such as lower intakes of fast foods and sweets by adolescents [50]. Larson et al. (2007), investigating FFM in adolescence and its relationship to food intake in young adulthood, showed a significant association of FFM with a higher quality diet, higher intakes of fruit, vegetables, and micronutrients, and lower intake of soft drinks [52]. Alamri (2020), examining the influence of the type of family meal (breakfast, lunch, and dinner) on the food intake of female adolescents, found that family breakfast was positively associated with the consumption of dairy products and proteins, family lunch was positively associated with eating vegetables and proteins, and family dinner was positively associated with the intake of fruit, vegetables, dairy products, and whole grains [17].

Leech et al. (2014) reported that daily family dinners were associated with healthy food habits, including higher consumption of fresh and dried fruits, vegetables, low-fat milk, and water and lower intake of energy-dense foods (sweet and savory foods, high-energy beverages), but only in male adolescents [54]. Lipsky et al. (2015) examined tendencies and changes in eating behaviors during the transition between adolescence and early adulthood and found that the intake of fruits, vegetables, and whole grains in this age range was positively associated with FFM [55]. Makansi et al. (2018) also evaluated eating habits and factors associated with fruit and vegetable intake, but no significant relationships were identified between FFM and fruit or vegetable consumption [57]. Martins et al. (2019) showed that eating at least 5 family meals per week was positively associated with the intake of beans, fruits, and vegetables (healthy diet score) and negatively associated with the consumption of sweets, ultra-processed salty foods, and fried salty snacks (unhealthy diet score) [58].

Neumark-Sztainer et al. (2003) assessed the patterns of family meals and their relationship to food and nutrient intake in a population of adolescents in the United States and found that adolescents with more frequent family meals had higher intakes of fruits, vegetables, grains, and calcium-rich foods. FFM was also negatively associated with the intake of soft drinks and snack foods [11]. Oliveira et al. (2018) reported that participants who ate more meals at home with their parents had a healthier diet and higher intakes of beans, vegetables, and milk, in addition to consuming smaller amounts of fried foods, sweets, and soft drinks on weekdays [59]. Totland et al. (2017) reported that lower frequency of family dinners was associated with lower vegetable intake among adolescents [60]. Walton et al. (2018) found that frequent family dinners were associated with healthier dietary habits, including higher fruit and vegetable intake and lower consumption of fast food and takeout in both boys and girls, as well as lower intake of sugar-sweetened beverages in boys [61].

Horning et al. (2016) identified a significant association of the frequency of family dinners with dietary quality and fruit and vegetable intake, as assessed by the Healthy Eating Index (HEI) [27]. Dietary quality and the intake of fruits and vegetables at dinner were also assessed by Arcan et al. (2019), who found that individuals with higher FFM were more likely to fill half their plate with fruits and vegetables, although they did not differ from their peers in terms of dietary quality [41]. Larson et al. (2016), investigating family meal experiences and fast-food purchasing among adolescents, evaluated several indicators of diet quality, but the only significant associations were found between the frequency of family breakfast and fruit intake in males, and refined grain intake in females [53]. Lipsky et al. (2017) showed that higher FFM was associated with higher scores on 3 diet quality indicators (HEI, Whole Plant Foods Density, and Empty Calories) [56]. Woodruff et al. (2009) showed that a higher frequency of family dinners was significantly associated with lower soft drink consumption and breakfast on the day of the study among adolescents [62]. Woodruff et al. (2010), investigating the association between FFM and overall diet quality, reported that adolescents who had 6 to 7 family meals per week had higher HEI scores than their peers [63]. Lastly, in another study, Woodruff et al. (2014) examined the association between family dinner frequency and number of meals and snacks per day and found that adolescents who had regular dinner with their families were likely to eat more meals and snacks on a daily basis as well as to consume fewer calories per day than those eating dinner with friends or alone [64].

## Discussion

FFM has been extensively explored in the literature. However, previous systematic reviews have addressed only some aspects of this topic, and the present study makes several novel contributions to the existing body of knowledge. We provide an update of previous reviews that is especially relevant given the large number of studies published between 2019 and 2020, as was the case of 8 articles included in the present review [17, 20, 22, 26, 41, 43, 51, 58]. This topic has been more extensively investigated in infants and toddlers due to the regularity of family meals in these age groups [65], but studies in older children are beginning to emerge as a result of the adoption of a holistic approach to determinants of health behaviors, in addition to the increased prevalence of noncommunicable diseases and overweight in this population [66]. The studies included in this review showed significant variations in the definition of regular and irregular family meal patterns, the type of meal evaluated, the time frame studied, and the response options available to participants. Additionally, most of the selected studies were conducted in the United States, and since some aspects of family meals differ among cultures [67] their findings may not apply to other populations. Nevertheless, all continents were represented in this review.

Despite the heterogeneity of findings across studies, the literature reviewed in the present study had several positive attributes, such as the absence of poor-quality studies, a high number of investigations involving large samples, including 33 studies of more than 1000 participants [11, 12, 18–22, 24, 25, 30, 32, 33–40, 44, 45, 47, 51, 52, 55, 58–63], the inclusion of boys and girls in almost all investigations, and the use of both cross-sectional and longitudinal designs in some studies. Several studies also investigated complementary variables and showed that family meals are influenced by other behavioral, psychological, and quality-of-life variables, such as household food security, physical activity levels of adolescents, and parental styles. These mediating factors should be explored in future studies, which will allow the measurement of their individual contribution to the phenomenon under study.

Of 25 studies evaluating the association between FFM and NS, 17 demonstrated an association between frequent family meals and lower incidence and prevalence of overweight in adolescents, suggesting that family meals may be protective against obesity in this population [12, 17, 21–31, 34–37]. However, some studies were sex-specific or ethnicity-specific, and these findings should also be explored in future reviews. Six studies did not find any significant association between the aforementioned variables [18–20, 32, 38–40], and only 2 of these studies [38, 39] also investigated dietary intake. A healthy diet is a main determinant of NS. Although family meals make a positive contribution to health by promoting commensality, slow chewing, bonding among family members, emotional and mental health, and even academic success by encouraging communication, their protective role against weight gain is heavily influenced by family eating habits; however, none of the included studies controlled for the influence of eating habits on the relationship between FFM and NS [6, 68]. Additionally, according to McCullough et al. (2016), the benefits of family meals are strongly influenced by the frequency, duration, and location of the meals, as well as by the type and amount of food offered, family communication, child or adolescent behavior, and the family members who are present, which underscores the importance of exploring additional variables pertaining to the family meal environment [69]. Moreover, the type of meal consumed with family (breakfast, lunch, and dinner or evening meals) might impact the FC of adolescents, since common foods consumed in each meal vary according to local culture. Alamri (2020) reported that different meals consumed with family were positively associated with the intake of different food groups [17]. In Brazil, for example, breakfast is usually associated with higher intakes of fruit and calcium, which can be explained by the traditional foods consumed in this meal [70]. Therefore, the relationship between the type of meal and FC warrants further investigation, including factors related to food culture.

Two studies revealed a significant association between FFM and weight gain in adolescents. One study [19] observed this association in Hispanic adolescents who were less acculturated and experienced lower parental control, suggesting that the influence of family meals on NS in adolescents also depends on factors such as parenting styles and the relationship between individuals and their home environment. This is in line with previous studies showing that having parents or guardians with low levels of behavioral control and high levels of authoritative parenting increases the risk of obesity in individuals from ethnic minorities, such as Hispanic children and adolescents living in the United States [71, 72]. The other study [33] showed an association of regular family meals with greater likelihood of overweight only in older children (6–11 years of age), highlighting the importance of further investigating this topic in future studies and addressing the issue of physical maturation in addition to the quality of the family diet.

An aspect that was not explored by the studies included in this review was the location of family meals. Eating meals outside the home or in fast-food restaurants, for instance, could contribute to weight gain and influence NS in adolescents. Therefore, in addition to FFM, it is important to investigate the quality and location of shared meals [7].

Studies investigating FC evaluated several healthy and unhealthy dietary practices and found that participants with higher FFM consumed more fruits, vegetables, whole grains, beans, and dairy products. In some studies, these individuals also showed a reduction in unhealthy dietary behaviors, such as the consumption of soft drinks, fried foods, and fast food. These findings are consistent with those of Hammons et al. (2011), who performed a meta-analysis of the frequency of shared meals and nutritional health in children and adolescents and included dietary intake as an outcome measure. The analysis showed that participants who shared meals with their families at least 3 times a week had healthier dietary habits and were 24% more likely to have healthier eating habits (OR = 1.24 CI: 1.13–1.37, p<0.01), such as higher fruit and vegetable intake and eating breakfast [73].

One study found a negative association between FFM and diet in adolescents [51], whereas 3 studies found no significant association between these variables [22, 48, 57]. Hong et al. (2019) reported that adolescents eating breakfast without their family consumed more milk and dairy products. Although this result appears negative, it can be explained by the typical South Korean breakfast, in which the foods typically consumed for breakfast are rice, kimchi, fish, and soup, among others, while the unhealthy breakfast pattern is usually milk, cereal, and industrialized versions of dairy products [51]. Assessment of dietary intake is complex, and clear operational definitions are required to ensure that the effects of exposure on this outcome are measured as accurately as possible. Other variables could also interfere with the association between FFM and dietary intake, such as family environment, eating environment, psychological factors, and watching television during meals. These elements should be considered in order to construct more reliable explanatory models and achieve a better comprehension of these phenomena. These factors may also be one of the reasons why the studies found no significant associations.

Four studies [27, 41, 56, 63] investigated dietary quality using original or adapted versions of the HEI and found healthy diet scores to be positively associated with FFM. This index was developed to assess overall diet quality, an approach that has been increasingly preferred over the assessment of individual attributes, such as the intake of specific food groups or nutrients [74]. It is interesting to note that some studies [46, 52, 55, 56] investigated associations between FFM in adolescence and eating habits in early adulthood, with significant findings for these variables. These results further support the role of family meals as a potentially positive influence on the adoption of healthier eating habits over the life course. One of these studies, conducted by Giray & Ferguson (2018), found that diet quality was not related to FFM but rather to the quality of the meal experience, revealing that other variables in addition to meal frequency can have a considerable influence on eating behaviors [48].

Although the literature points to a positive association of family meals with quality of diet and healthy NS, there is still no consensus on the ideal number of meals, type of meal, or other meal patterns that families should adopt to really make it a protective factor for nutritional health in adolescents. Some studies have already provided important clues about this positive association, such as the study by Tosatti et al. (2017) [9], who reinforced the need to expand investigations by using designs that allow establishing such parameters. It will be useful to advise and guide families on this subject and fill the gaps of previous research. It is also essential to consider the cultural peculiarities that influence FC and eating practices around the world.

## Conclusions

This review showed an association between FFM and healthy dietary patterns, such as increased consumption of fruits, vegetables, whole grains, and beans. Further research is needed to understand the association between FFM and NS, since some studies showed a protective role of family meals against obesity in this age group, whereas other studies identified no significant association between these variables. Nevertheless, a healthy diet is a known determinant of NS, and most studies of the association between FFM and NS did not control for FC, which may have influenced their findings. Future research examining associations between FFM and FC and/or NS should consider other meal characteristics such as the duration and location of the meals, the type of meal, and the type and/or amount of food served.

## Supporting information

S1 Fig PubMed search strategy. (TIF)

S2 Fig Study selection process. (TIFF)

S1 File Data extraction table of included studies. (XLSX)

S2 File Risk of bias of included studies. (XLSX)

S3 File PRISMA checklist. (XLSX)

S1 Data (XLSX)

S1 Checklist (PDF)
