# Supplementary figures and images for: Family meal frequency and its association with food consumption and nutritional status in adolescents: A systematic review
Source: PLoS One. 2020 Sep 18;15(9):e0239274. doi: 10.1371/journal.pone.0239274 (PMC7500660; doi:10.1371/journal.pone.0239274)

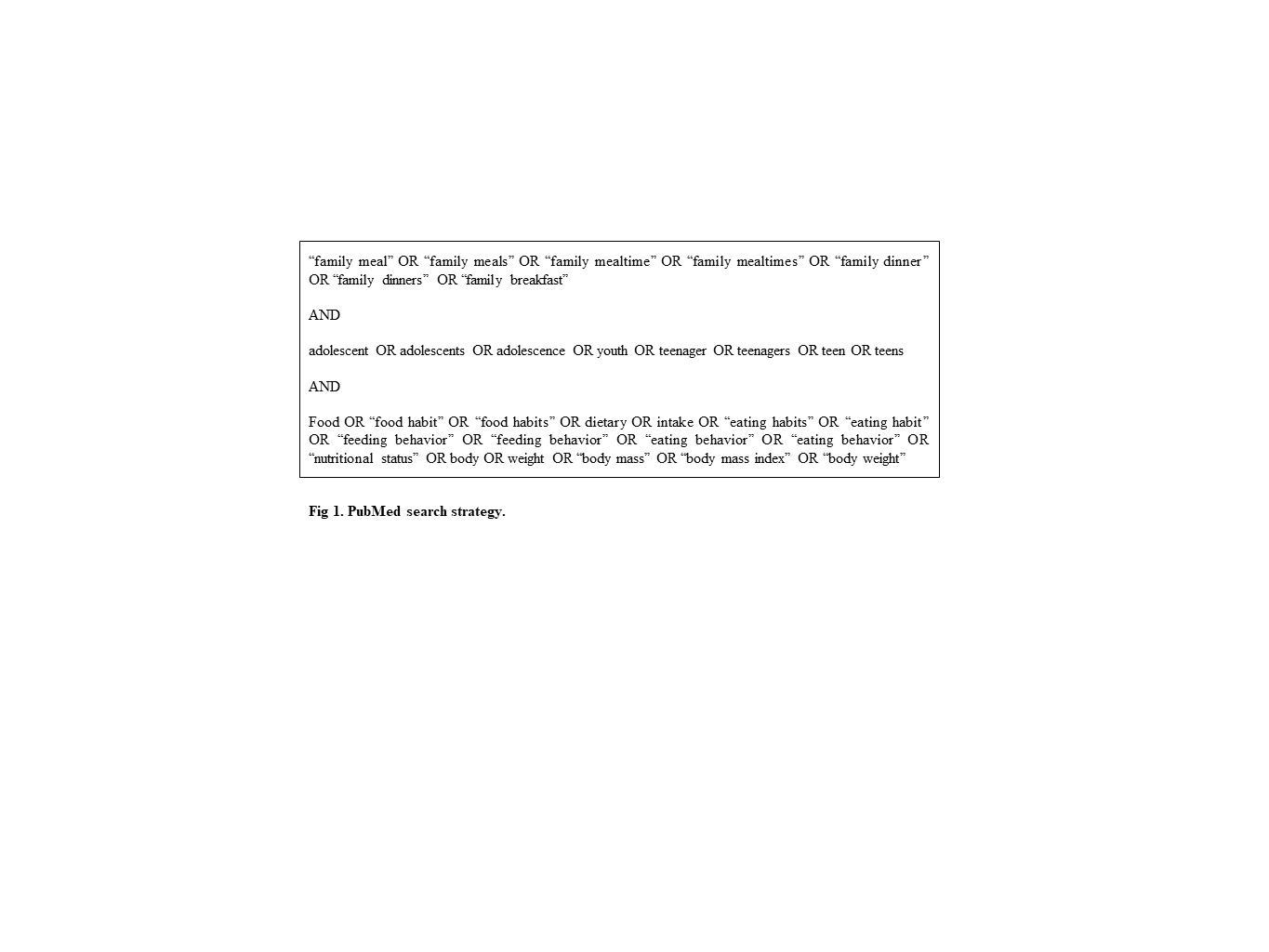

Supplement: S1 Fig — (TIF) [file pone.0239274.s001.tif]

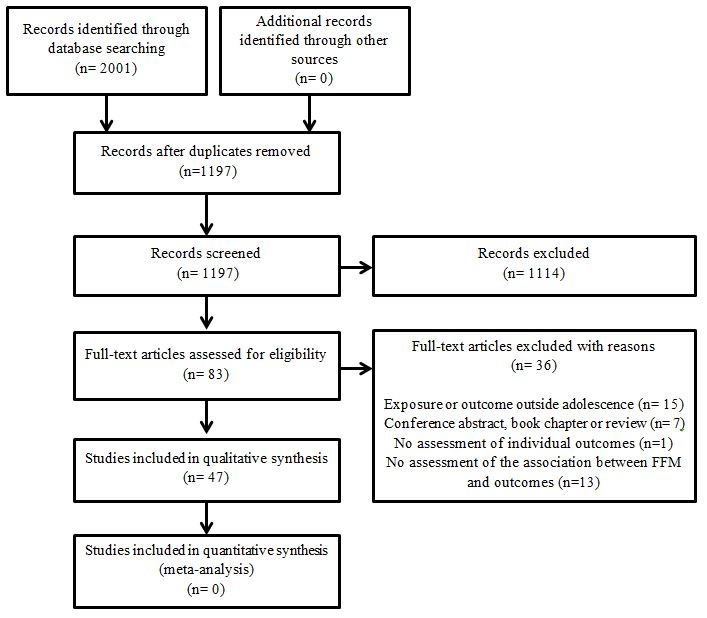

Supplement: S2 Fig — (TIFF) [file pone.0239274.s002.tiff]
